# Gastric Perforation Associated with Tuberculosis: A Case Report

**DOI:** 10.1155/2011/392769

**Published:** 2011-05-05

**Authors:** Richdeep S. Gill, Sumeet S. Gill, Harshdeep Mangat, Sarvesh Logsetty

**Affiliations:** ^1^Department of Surgery, University of Alberta, 9-50 Medical Sciences Building, Edmonton, AB, Canada T6G 2N8; ^2^Faculty of Kinesiology, University of Calgary, Calgary, AB, Canada T2N 1N4; ^3^Faculty of Medicine and Dentistry, University of Alberta, Edmonton, AB, Canada T6G 2R7; ^4^Department of Surgery, The University of Manitoba, Winnipeg, MB, Canada R3B 2E9

## Abstract

Gastric tuberculosis is a rare presentation of tuberculosis infection. Gastric perforation associated with tuberculosis is exceedingly rare with five previously published cases. We present a case of a male patient that developed presumed gastric tuberculosis secondary to pulmonary tuberculosis infection. He subsequently developed gastric perforation and sepsis, for which he was treated both surgically and medically. Despite ongoing antituberculosis treatment, the patient's condition worsened and the patient died secondary to multiorgan failure. This case highlights gastric perforation as a rare but devastating complication of pulmonary tuberculosis.

## 1. Introduction

Gastric tuberculosis is a rare presentation of tuberculosis (TB) infection [1] with a reported incidence of less than 0.2% on routine gastric biopsies [2]. However, the frequency of gastric tuberculosis increases dramatically to 4.5% in individuals with moderate pulmonary disease and 25% in those with severe disease [3]. Regardless of whether gastric tuberculosis is primary or secondary, gastric perforation is exceedingly rare with 5 previous cases reported in the literature [4]. We present a case of a patient with pulmonary tuberculosis complicated by a large gastric perforation.

## 2. Case Report

A 57-year-old HIV-negative aboriginal male was initially admitted with community-acquired pneumonia, which progressively worsened. A subsequent bronchoscopy with bronchoalveolar lavage revealed acid-fast bacilli on ziehl-neelsen staining. A subsequent CT chest demonstrated bilateral pulmonary infiltrates and cavitating lesions consistent with active pulmonary tuberculosis infection. His treatment was initiated with the standard 4-drug regimen: pyrazinamide, rifampin, ethambutol, and isoniazid (INH). Sputum mycobacterial TB culture demonstrated susceptibility to the 4-drug regimen. 

During his admission to hospital, he developed diffuse abdominal pain with peritonitis on examination. He also developed tachycardia and hypotension, and an abdominal X-ray revealed free air under the diaphragm. Arrangements were made for emergent exploratory laparotomy in the operating room. Intraoperatively a single contiguous inflammatory mass was found in the left upper quadrant, which revealed a gangrenous necrotic splenic colon. A left hemicolectomy was preformed. This revealed a region of necrotic-appearing pancreatic tissue which was likely associated with the necrosis of the adjacent splenic colon. Further exploration of this inflammatory mass demonstrated particulate matter and peritoneal fluid and a large perforation in the posterior wall of the stomach, which was the initial insult. Peritoneal fluid mixed with enteric contents from the gastric perforation was sent for culture and was positive for mycobacterium tuberculosis. An intraoperative gastroscopy was preformed which identified an ulcer approximately 5–10 cm from the gastroesophageal junction. The patient hemodynamic status worsened in the operating room despite ongoing resuscitation and significant quantities of norepinephrine intravenously were needed. It was decided to stabilize the patient with a damage control laparotomy and take the patient to the intensive care unit rather than perform a definitive repair of the gastric perforation because of the severe hemodynamic instability. Drains surrounding the ulcer were placed and the skin closed. Plans were made to return to the operating room in 24 hours for definitive treatment if the patient's hemodynamic status improved. 

The patient's condition stabilized in the intensive care unit, and he was brought to the operation room the following day. Multiple biopsies of the large 3 cm gastric ulcer were taken. The patient was deemed to be unable to tolerate a total gastrectomy at this time, and unfortunately wedge resection of the stomach was not possible due to the position and size of the ulcer. Thus, the ulcer was repaired with primary closure, and creation of a feeding jejunostomy was completed. The patient was returned to the intensive care unit. Biopsies from the gastric ulcer revealed no malignancy but did demonstrate fungal elements (Candida albicans) indicative of secondary infection. Also, focal reactive changes in the surface epithelium suggested a reactive process. No granulomata or giant cells were identified. Biopsies were also negative for Helicobacter pylori. The patient did not receive nonsteroidal anti-inflammatory medications and was on Pantoprazole to prevent stress-related ulceration. The peritoneal fluid cultures collected during operation for the gastric perforation demonstrated mycobacterium tuberculosis and fungal elements. Gastric tissue sent intraoperatively for culture was positive for mycobacterium TB, which led to the presumptive diagnosis of gastric perforation, associated with TB. Pathologic examination of the resected left colon revealed an area of necrosis, but no perforation. Microscopic examination demonstrated numerous caseating granulomas with multinucleated giant cells, demonstrating mycobacterial colitis. 

The patient remained in the intensive care unit, and a small-bowel follow-through with contrast revealed no extravasation of contrast. The TB-associated gastric perforation was adequately repaired. Postoperatively the patient was continued on INH 300 mg intramuscularly and rifampin 600 mg intravenously daily according to sensitivities from peritoneal fluid cultures. Imipenum, levofloxacin, and fluconazole were also continued according to culture results. 

The patient's pulmonary tuberculosis persisted over the subsequent weeks despite appropriate antituberculosis treatment. Deteriorating lung function was likely a combination of previous smoking damage, edema secondary to recent aggressive fluid resuscitation, and persistent pneumothorax. Eventually multiorgan failure ensued, and life support was discontinued following discussion with the family.

## 3. Discussion

An estimated 3 million deaths worldwide are attributed to TB each year [5]. TB may affect any part of the gastrointestinal tract [6] with the ileocecal region being most commonly involved. Gastric TB, however, is comparatively rare [7]. The low incidence of gastric TB is postulated to be due to the scarce presence of lymphoid tissue in the stomach combined with the presence of an acidic environment [1]. Hence the prepyloric region of the stomach seems to be the most common segment involved because of the presence of lymph follicles [8]. 

Gastric TB may originate primarily within the stomach [6], but it is most commonly secondary to preexisting pulmonary infection. The patient develops gastric TB secondary to swallowed organisms [9]. Also, gastric TB may develop from other GI sites due to hematogenous spread [10] or spread from adjacent celiac nodes [9], with the most common location for gastrointestinal TB being the ileocecal region [11]. Interestingly, the presence of TB in the stomach secondary to swallowed pulmonary TB may be relatively common. In children, the use of gastric lavage has been utilized to diagnose TB [12]. A majority of the patients were outpatients in this systematic review. Thus, the presence of TB from swallowed sputum only leads to gastric TB in rare cases. 

In five previously reported cases of gastric perforation, four were diagnosed with TB by the presence of acid-fast bacilli on gastric biopsy or the presence of lymph nodes with caseating necrosis [4, 13, 14]. Similar to our case, one of the reported cases in this review had nonspecific inflammation on gastric biopsy. Diagnosis of TB in this previous case was based on the presence of acid-fast bacilli in the lymph nodes [15]. In our patient, no lymph nodes were biopsied intraoperatively, rather enteric contents and peritoneal fluid within the abdominal cavity and gastric biopsy specimen sent for culture were positive for mycobacterium tuberculosis. However, these positive cultures of the gastric specimen and peritoneal fluid may be secondary to swallowed sputum. Despite this possibility, after considering alternative diagnoses, gastric perforation associated with TB remains the most likely.

Appropriate surgical management gastric perforation is dependent on the location and size of the perforation. In the previous five published cases of gastric perforation was treated with distal or total gastrectomy [4]. In our patient case, his continued sepsis and hemodynamic instability in the previous operation led us to consider a more conservative approach. With the stomach appearing generally healthy despite the perforation, this approach proved reasonable and successful. However, gastrectomy would seem the ideal option because it would remove the infectious process in the involved organ. 

Antituberculosis treatment is the mainstay of therapy of uncomplicated infection diagnosed by endoscopic biopsy [16]. Complicated gastrointestinal infections such as TB-associated gastric perforation have significant mortality [4]. Of the six reported cases of TB-associated gastric perforation, only one patient has survived with prompt surgical intervention and anti-TB therapy [4]. In our patient, despite successful operative repair of the gastric perforation, multiorgan failure secondary to pulmonary TB resulted in mortality. TB is a potentially devastating infection that is rarely seen and even more rarely in Canada. This case highlights gastric perforation as a rare but devastating complication of pulmonary tuberculosis.
